# Central Mechanism Controlling Pubertal Onset in Mammals: A Triggering Role of Kisspeptin

**DOI:** 10.3389/fendo.2019.00312

**Published:** 2019-05-21

**Authors:** Yoshihisa Uenoyama, Naoko Inoue, Sho Nakamura, Hiroko Tsukamura

**Affiliations:** ^1^Laboratory of Animal Reproduction, Graduate School of Bioagricultural Sciences, Nagoya University, Nagoya, Japan; ^2^Faculty of Veterinary Medicine, Okayama University of Science, Imabari, Japan

**Keywords:** dynorphin A, gonadotropin-releasing hormone (GnRH), GnRH pulse generator, kisspeptin, KNDy, neurokinin B, puberty

## Abstract

Pubertal onset is thought to be timed by an increase in pulsatile gonadotropin-releasing hormone (GnRH)/gonadotropin secretion in mammals. The underlying mechanism of pubertal onset in mammals is still an open question. Evidence accumulated in the last 15 years suggests that kisspeptin/neurokinin B/dynorphin A (KNDy) neurons in the hypothalamic arcuate nucleus play a key role in pubertal onset by triggering pulsatile GnRH/gonadotropin secretin in mammals. Specifically, KNDy neurons are now considered a part of GnRH pulse generator, in which neurokinin B facilitates and dynorphin A inhibits, the synchronized discharge of KNDy neurons in autocrine and/or paracrine manners. Kisspeptin serves as a potent secretagogue of GnRH secretion and thus its release is fundamental to pubertal increase in GnRH/gonadotropin secretion in mammals. Proposed mechanisms inhibiting *Kiss1* (kisspeptin gene) expression during childhood to juvenile varies from species to species: we envisage that negative feedback action of estrogen plays a key role in the inhibition of *Kiss1* expression in KNDy neurons in rodents and sheep, whereas estrogen-independent inhibition of kisspeptin secretion by γ-amino butyric acid or neuropeptide Y are suggested to be responsible for the pre-pubertal suppression of GnRH/gonadotropin secretion in primates. Taken together, the timing of pubertal onset is postulated to be controlled by upstream regulators for kisspeptin biosynthesis and secretion in mammals.

## Introduction

The reproductive system is governed by the hypothalamo-pituitary-gonadal axis and has a unique functional quiescence during childhood in mammals. Pubertal onset is thought to be timed by an increase in gonadotropin-releasing hormone (GnRH)/gonadotropin secretion. The underlying mechanism controlling pubertal onset still remains elusive (1). Evidence accumulated in the last 15 years suggests that a neuropeptide kisspeptin plays a key role in pubertal onset via triggering GnRH/gonadotropin secretion in mammals. This article provides a brief historical background including the discoveries of gonadotropins, GnRH, and kisspeptin, and our current understanding of how the brain controls pubertal onset in mammals.

## GnRH/Gonadotropin Secretion Triggers Pubertal Onset in Mammals

The concept that factor(s) synthesized and secreted by the pituitary gland controls pubertal onset in mammals dates back to the late 1920s, when physiologists examined effects of hypophysectomy or extract of the pituitary gland on gonadal activities and found the factor(s) that induced pubertal onset in immature rodents (2). First, they believed that the pituitary gland synthesizes and secretes a single gonadotropic factor. Later, increasing evidence suggested the existence of two gonadotropic factors in the pituitary gland and two gonadotropins, follicle-stimulating hormone (FSH) and luteinizing hormone (LH), were successfully purified from the pituitary gland in the early 1930s (3). In the late 1940s, Harris predicted that the secretion of pituitary gonadotropins would be controlled by factor(s) secreted from the hypothalamic neurons through the pituitary portal circulation (4). This prediction promoted studies on the discovery of such hypothalamic factor(s) in several mammals. Consequently, the mammalian form of GnRH, a decapeptide, which stimulates both FSH and LH secretion from the pituitary gland, was isolated and sequenced by two independent laboratories led by Schally and Guillemin in the early 1970s (5, 6).

Establishment of the radioimmunoassay by Yalow and Berson (7) in 1950s has facilitated studies on GnRH/gonadotropin secretion. By using frequent blood collection and this radioimmunoassay, Knobil et al. clearly demonstrated that two modes of gonadotropin secretion in female rhesus monkeys, as a model of humans (8–10): one is tonic gonadotropin secretion that controls follicular development and steroidogenesis in the ovary and the other is a large secretion of LH, namely a LH surge, that controls ovulation and corpus luteum formation. As for tonic secretion, Knobil et al. successfully showed the pulsatile nature of LH secretion (9). They assumed that the pulsatile nature of tonic gonadotropin secretion is most likely caused by pulsatile GnRH stimulation to the pituitary gland and clearly demonstrated the physiological significance of pulsatile GnRH stimulation on the pituitary gland to maintain circulating gonadotropin levels (11). Importantly, continuous GnRH stimulation to the pituitary gland suppressed gonadotropin secretion (11). These findings clearly indicate that the pulsatile nature of GnRH secretion is required to maintain the normal responsiveness of the pituitary gland to GnRH. The Knobil laboratory also demonstrated that this pulsatile GnRH stimulation successfully triggered pubertal onset in immature female monkeys (12). This finding suggests that an initiation of pulsatile GnRH secretion is the first step in pubertal onset in mammals. Accumulating evidence suggests that pubertal onset is triggered by an increase in pulsatile secretion of GnRH/gonadotropins in several mammalian species (13–16).

Pulsatile GnRH secretion in the pituitary portal circulation was first described in sheep in 1982 by Clarke and Cummins (17) with a pituitary portal cannulation, and then examined with the same skillful technique in more detail in 1992 by Moenter et al. (18). These studies clearly demonstrated that each GnRH pulse detected in the pituitary portal circulation corresponds to each LH pulse detected in the peripheral circulation. Pulsatile GnRH secretion is not easily detectable in the peripheral circulation due to a combination of the short half-life of GnRH and the dilution in the relatively large volume of peripheral circulation (18). Therefore, plasma LH profiles showing pulsatile LH secretion have been used as good surrogate for pulsatile GnRH secretion into the pituitary portal circulation in mammals.

## Kisspeptin Controls GnRH Pulse Generation and Pubertal Onset

Pulsatile GnRH secretion from the nerve terminals of GnRH neurons at the median eminence into the pituitary portal circulation has been assumed to be driven by a so-called “GnRH pulse generator” (19, 20). The Knobil laboratory first established a method for the detection of GnRH pulse generator activity via an electrophysiological approach in rhesus monkeys (21): They clearly demonstrated that rhythmic increases in the multiple unit activity (MUA), correspond to LH pulses detected in the peripheral circulation, was successfully monitored from the recording electrodes implanted in the mediobasal hypothalamus. This electrophysiological approach was subsequently adapted to rats and goats (22–24) and showed that rhythmic increases in the MUA recorded in the mediobasal hypothalamus, or more specifically in the arcuate nucleus (ARC), also correspond to LH pulses in such species.

Increasing evidence suggests that hypothalamic kisspeptin [first named as metastin (25)] neurons play a key role in controlling pubertal onset via stimulation of GnRH/gonadotropin secretion in mammals. A novel peptide kisspeptin was discovered from the human placenta as an endogenous ligand of GPR54, an orphan G-protein coupled receptor, in 2001 (25, 26). Subsequently, clinical studies revealed that inactivating mutations of the *GPR54* gene caused the impairment of pubertal maturation and reproductive functions with hyposecretion of gonadotropins, i.e., hypogonadotropic hypogonadism, in humans (27, 28). These findings suggest that kisspeptin serves as a gatekeeper of pubertal onset in the hypothalamo-pituitary cascade regulating reproductive system in humans. Indeed, kisspeptin profoundly stimulated gonadotropin secretion in several mammals including humans (29–35) and the stimulatory effect of kisspeptin on gonadotropin secretion was blocked by GnRH antagonists in rodent models (29–31). These studies clearly indicate a physiological role of kisspeptin as a potent secretagogue of GnRH in the hypothalamus. In 2012, the phenotypes of patients carrying *GPR54* mutations, i.e., hypogonadotropic hypogonadism and lack of puberty, was recapitulated in patients carrying inactivating mutations of the *KISS1* gene (coding kisspeptin) (36). Further, several rodent models lacking functional kisspeptin or its receptor by gene targeting, namely *Kiss1* or *Gpr54* knockout mice, showed pubertal failure and infertility (28, 34, 37–39). In addition, *Kiss1* knockout rats never showed puberty and demonstrated a lack of both pulse and surge modes of gonadotropin secretion (40). These findings strongly suggest that kisspeptin-GPR54 signaling plays a key role in the mechanism controlling GnRH/gonadotropin secretion and pubertal onset in mammals. Indeed, Keen et al. (41) demonstrated that a pubertal increase in pulsatile kisspeptin secretion along with pulsatile GnRH secretion at the median eminence in rhesus monkeys. Kisspeptin administration successfully stimulated GnRH/LH secretion in pre-pubertal and pubertal mammals including rodents (42–44) and primates (45–47).

It is well-accepted that kisspeptin directly stimulates GnRH secretion via GPR54 expressed in GnRH neurons in rodents (30, 34). Indeed, the targeted deletion of *Gpr54* in GnRH neurons of mice resulted in infertility, whereas the reintroduction of *Gpr54* to GnRH neurons in *Gpr54*-null mice resulted in fertility (48), suggesting that GPR54 expression solely in GnRH neurons is sufficient for fertility. A previous study showed stable expression of GnRH mRNA in the hypothalamus during the pubertal transition in rats (49). Thus, kisspeptin has a role to trigger GnRH secretion rather than GnRH synthesis at the onset of puberty in mammals.

## Two Populations of Kisspeptin Neurons Control Pulse and Surge-Mode of GnRH Secretion

There are two populations of hypothalamic kisspeptin neurons: one is localized in the ARC and the other population is localized in the anteroventral periventricular nucleus and periventricular nucleus continuum (AVPV-PeN) in rodents and the pre-optic area (POA) in other mammals including ruminants, primates, pigs, and musk shrews (50–60). Kisspeptin neurons localized in the AVPV-PeN of rodents are possibly equivalent to those in the POA of the other animals. Accumulating evidence suggests that the neurons in the ARC have a role in GnRH pulse generation described above, whereas the neurons in the AVPV-PeN have been recognized to play a pivotal role in mediating positive feedback action of estrogen to induce GnRH/LH surge in rodents (61–64).

**Figure 1:**
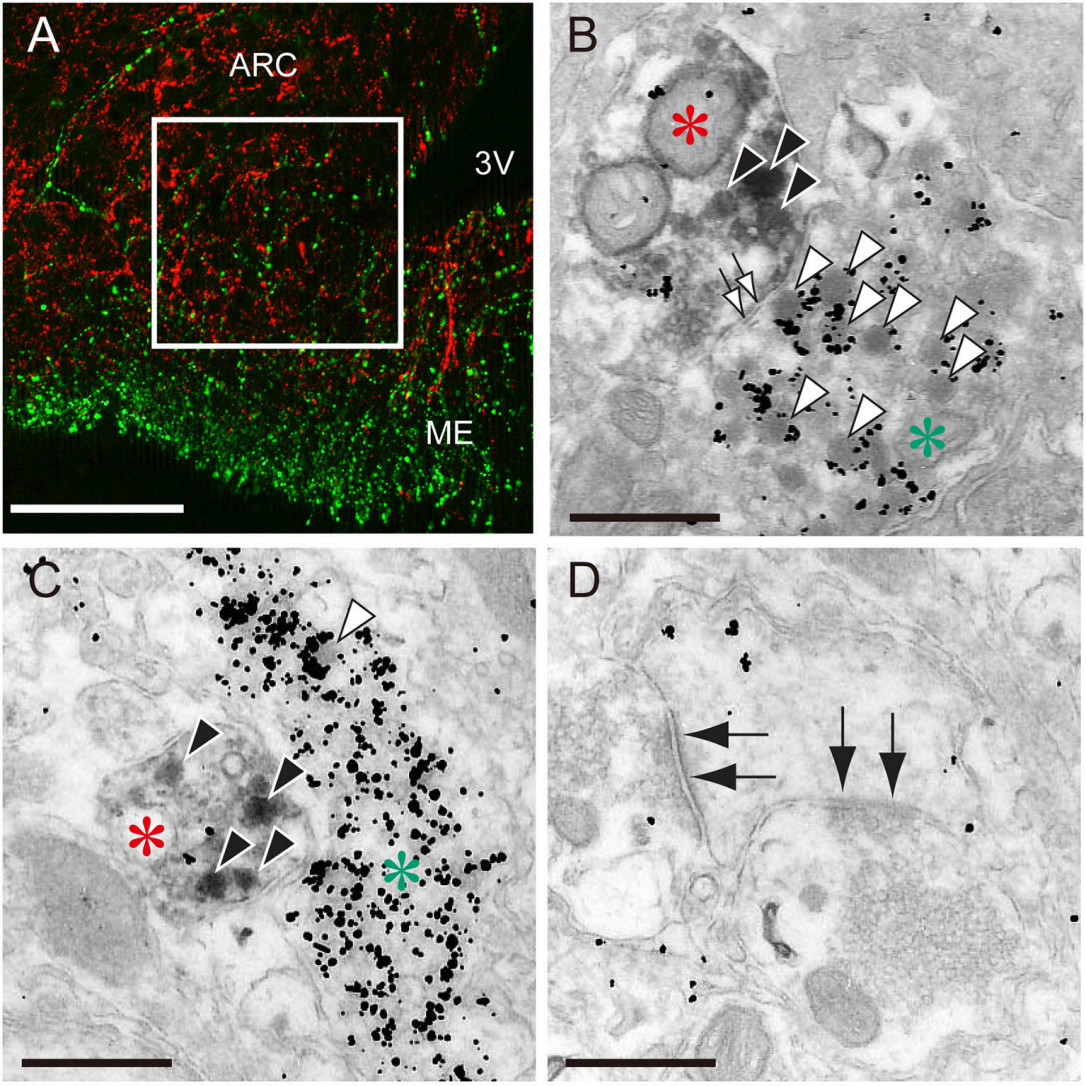
The close association of kisspeptin and gonadotropin-releasing hormone (GnRH) fibers in the median eminence of female rats. **(A)** An immunofluorescence photomicrograph showing kisspeptin (red)- and GnRH (green)-immunoreactivities in the arcuate nucleus (ARC)-median eminence (ME) region. Note that the square in the internal zone of the median eminence shows the area for electron microscopy. Scale bar, 50 μm. 3V, third ventricle. **(B,C)** Immunoelectron micrographs showing kisspeptin- (red asterisks) and GnRH-immunoreactive fibers (green asterisks) visualized by diaminobenzidine and silver enhancement of colloidal gold, respectively. Solid and open arrowheads show kisspeptin- and GnRH-immunoreactive secretory granules, respectively. Open arrows show non-synaptic contact between kisspeptin- and GnRH-neuronal elements. Scale bar, 500 nm. **(D)** Electron micrograph showing synaptic contacts (solid allows) in other neurons. Scale bar, 500 nm. Originally published in Uenoyama et al. (66) (permission was obtained from John Wiley and Sons).

The most plausible interpretation is that ARC kisspeptin neurons serve as a part of the GnRH pulse generator in mammals, because rhythmic increases in the MUA are successfully detected from recording electrodes that are placed in close proximately to ARC kisspeptin neurons in goats (53) but not in the lateral median eminence where GnRH nerve terminals are abundantly located. Further, a study with a GCaMP6 fiber photometry technique demonstrated in mice that ARC kisspeptin neurons exhibited rhythmic increases in intra-cellular Ca^2+^ corresponding to LH pulses and that optogenetic stimulation or inhibition of ARC kisspeptin neurons induced or suppressed pulsatile LH secretion, respectively (65). GnRH neuronal terminals in the median eminence seem to be an action site of kisspeptin for the generation of GnRH pulses (Figure 1), because immunoelectron microscopy revealed a close association of kisspeptin and GnRH fibers in the median eminence in rats and goats (66, 67). Further, few typical synaptic structures between kisspeptin and GnRH fibers were found in the median eminence, suggesting that kisspeptin acts on GnRH fibers in a non-synaptic manner, such as “volume transmission” (66, 67). GnRH cell bodies also seem to be an action site of ARC kisspeptin neurons, because a tract-tracing study revealed the projection of ARC kisspeptin neuron to the POA in mice (68) and confocal microscopy revealed contacts of kisspeptin fibers from the ARC population to GnRH neurons in ewes (69).

Increasing evidence suggests that the anterior population of hypothalamic kisspeptin neurons serves as GnRH surge generator that evokes the GnRH/LH surge in response to the positive feedback action of estrogen derived from the pre-ovulatory follicles in mammals, because estrogen up-regulates *Kiss1* expression and/or neuronal activation in this population (52, 54–57, 70–72). We recently reviewed the sexual dimorphism and role of AVPV-PeN kisspeptin neurons elsewhere (73).

In contrast to AVPV *Kiss1* expression, ARC *Kiss1* expression is down-regulated by estrogen. This suggests that ARC kisspeptin neurons mediate the negative feedback action of estrogen on pulsatile gonadotropin secretion in mammals (33, 50, 52). Recently, Treen et al. (74) have generated immortalized *Kiss1*-expressing cells from the murine hypothalamus and successfully replicated estrogenic regulation of *Kiss1* expression in both AVPV- and ARC-derived immortalized *Kiss1*-expressing cells. Such immortalized AVPV and ARC *Kiss1*-expressing cells would serve as a promising platform to explore the molecular mechanism mediating estrogenic controls of *Kiss1* expression in the two hypothalamic kisspeptin neurons.

Interestingly, the interaction between ARC and AVPV kisspeptin neurons was recently suggested through a tract-tracing study showed that ARC kisspeptin neurons project to the AVPV kisspeptin neurons in mice (75). Further, an optogenetic study indicated that the channelrhodopsin-2-mediated light stimulation of ARC kisspeptin neurons activated GnRH neurons via AVPV kisspeptin neurons and suggested that this pathway is likely involved in the pre-ovulatory GnRH/LH surge (76). This finding is consistent with our previous finding that ARC kisspeptin neurons are activated at the proestrous stage of estrus cycle in female rats (33). Interestingly, *Kiss1* expression in both POA and caudal ARC increased just before GnRH/LH surges in ewes and monkeys (59, 60, 77). These findings are also consistent with previous findings that the mediobasal hypothalamus including the ARC is likely involved in GnRH/LH surge generation in those species (78–81).

Ours and other previous studies showed a pubertal increase in both ARC and AVPV *Kiss1* expression in female rodents (42, 51, 82). As discussed below, we envision that the regulation of ARC *Kiss1* expression is fundamental to control pubertal onset of tonic gonadotropin secretion, whereas AVPV *Kiss1* expression is secondarily controlled by estrogen secreted from the ovary under the tonic stimulation of gonadotropin (82).

## Cellular and Molecular Mechanism of GnRH Pulse Generation and Pubertal Onset

Clinical studies also demonstrated the involvement of neurokinin B, a member of tachykinin family (83), in regulation of GnRH/gonadotropin secretion and pubertal onset in humans: loss-of-function mutations of *TAC3* gene (coding neurokinin B) or *TACR3* gene (coding tachykinin NK3 receptor (NK3R), which preferably bind to neurokinin B) also caused hypogonadotropic hypogonadism and pubertal failure in humans (84–86). It is noteworthy that both neurokinin B and NK3R are co-localized in most of ARC kisspeptin neurons of several mammalian species (87–89). Further, dynorphin A, an endogenous opioid peptide, is also co-localized in ARC kisspeptin neurons (87–89). Thus, the kisspeptin/neurokinin B/dynorphin A (KNDy) co-expressing neurons in the ARC are referred to as KNDy neurons (90). Wakabayashi et al. (89) clearly demonstrated that a central administration of neurokinin B facilitated the frequency of the rhythmic increases in the MUA in goats. They also demonstrated that the frequency of the rhythmic increases in the MUA was suppressed by a central administration of dynorphin A and facilitated by antagonism of the kappa-opioid receptor (KOR, which preferably binds to dynorphin A) signaling in goats (89). Similarly, a central administration of neurokinin B or KOR antagonist facilitated pulsatile LH secretion in sheep (91, 92). Recently, Weems et al. demonstrated that KOR is expressed in both KNDy and GnRH neurons and proposed a current working model that dynorphin A acts on both KNDy and GnRH neurons to terminate each GnRH pulse in ewes (93–95). This is in agreement with our previous study that showed that chronic peripheral administration of NK3R agonist or KOR antagonist prior to puberty advanced pubertal onset in female rats via an increase in tonic gonadotropin secretion (96). Taken together, it is tempting to speculate that neurokinin B-NK3R and/or dynorphin A-KOR signaling may serve as a driving force for GnRH pulse generation to trigger the pubertal onset in an autocrine and/or paracrine manner in mammals. Previous studies have shown pubertal increase in ARC *Tac3* as well as *Kiss1* expression in mRNA or protein levels in rodents (82, 97, 98) and sheep (99).

**Figure 2:**
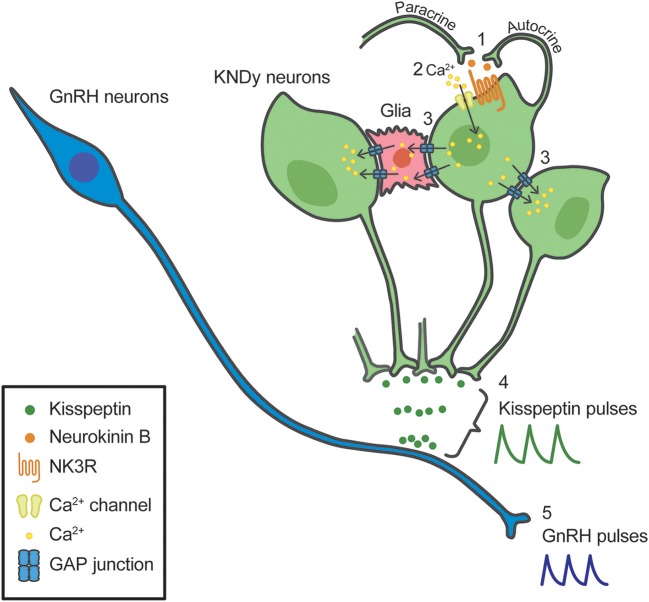
Schematic illustration of a model for the synchronized discharge of arcuate kisspeptin/neurokinin B/dynorphin A (KNDy) neurons and subsequent GnRH secretion. Neurokinin B secreted from KNDy neurons (green) binds tachykinin NK3 receptor (NK3R) in an autocrine/paracrine manner **(1)**, triggering Ca^2+^ influx into the NK3R-expressing KNDy neurons **(2)**. The increased intracellular Ca^2+^ may propagate to neighboring KNDy neurons and glial cells (red) via gap junctions, even if those neurons do not express NK3R **(3)**. The propagation of intracellular Ca^2+^ increase would result in synchronized discharge of KNDy neurons and then causes pulsatile kisspeptin secretion **(4)**. Resultant pulsatile kisspeptin secretion controls pulsatile GnRH secretion at the median eminence **(5)**.

Our recent *in vitro* study using a primary culture of green fluorescent protein (GFP)-tagged kisspeptin neurons obtained from transgenic *Kiss1*-GFP mice, proposed possible involvement of gap junctions between KNDy neurons as well as between KNDy neurons and glial cells in the generation of rhythmic activity of kisspeptin neurons under the neurokinin B-NK3R signaling (100). Briefly, neurokinin B secreted from KNDy neurons binds NK3R to open Ca^2+^ channels in an autocrine/paracrine manner (#1 in Figure 2), triggering Ca^2+^ influx into the NK3R-expressing KNDy neurons (#2 in Figure 2). The increased intracellular Ca^2+^ may propagate to neighboring KNDy neurons and glial cells via gap junctions, even if those neurons do not express NK3R (#3 in Figure 2). The gap junctions between KNDy neurons or between KNDy neurons and glial cells might be formed, at least in part, by connexins 26 or 37, because quantitative RT-PCR analysis showed *Gjb2* (connexin 26 gene) and *Gja4* (connexin 37 gene) expression in kisspeptin neurons of the *Kiss1*-GFP mice (100). The propagation of intracellular Ca^2+^ increase would result in synchronized discharge of ARC KNDy neurons and then causes pulsatile kisspeptin secretion at the median eminence (#4 in Figure 2). Finally, kisspeptin in turn induces pulsatile GnRH secretion into the pituitary portal circulation (#5 in Figure 2). Neurokinin B-NK3R signaling is supposed to be a major mechanism underlying the synchronized discharge of KNDy neurons, because ~80% KNDy neurons express NK3R (100, 101). The gap junctional communication would contribute to ensure synchronized discharge of KNDy neurons regardless of NK3R expression. Indeed, a previous study reported that patients with mutations of connexin-26 showed hypogonadotropic hypogonadism and a lack of pubertal onset (102).

## Species Difference in the Pre-Pubertal Restraint of GnRH/Gonadotropin Secretion

**Figure 3:**
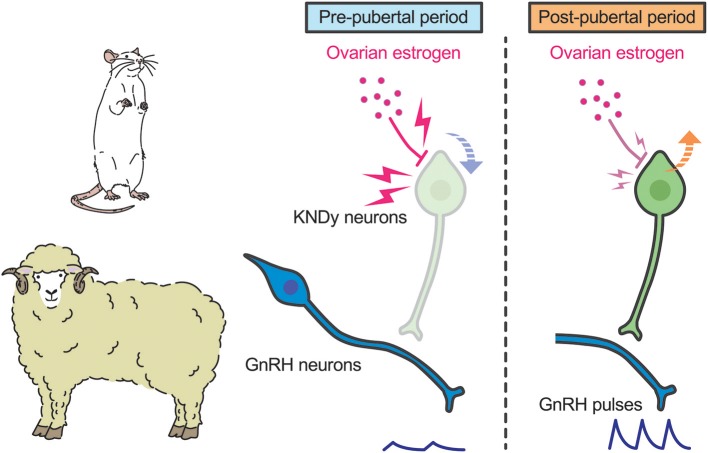
Schematic illustration showing a current interpretation for the brain mechanism controlling pubertal onset in rats and sheep. We envision that ovarian estrogen strongly suppresses ARC *Kiss1* expression **(down arrow)**. The inhibitory action of estrogen on ARC *Kiss1* expression somehow decreases during the pubertal transition, resulting in an increase in *Kiss1* expression **(up arrow)**. Resultant pulsatile kisspeptin secretion triggers pubertal increase in pulsatile GnRH secretion.

**Figure 4:**
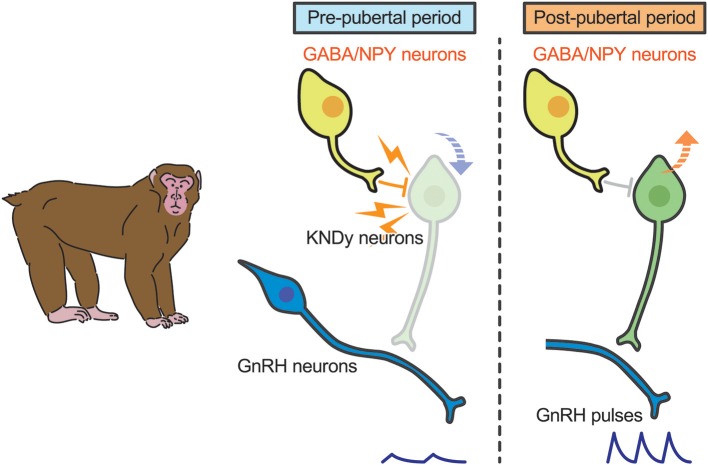
Schematic illustration showing a current interpretation for brain mechanism controlling pubertal onset in primates. We envision that γ-amino butyric acid (GABA) or neuropeptide Y (NPY) suppresses kisspeptin secretion **(down arrow)** in primates. Those inhibitory actions somehow disappear during the pubertal transition, resulting in an increase in *Kiss1* expression **(up arrow)**. Resultant kisspeptin secretion triggers pubertal increase in GnRH secretion.

A current interpretation for brain mechanism controlling pubertal onset in mammals is presented schematically in Figures 3, 4. Several lines of evidence suggest that the key players in this mechanism are ARC KNDy neurons, which serve as the GnRH pulse generator, regulate pulsatile GnRH/gonadotropin secretion and hence pubertal onset in mammals including rodents, ruminants, and primates. GnRH pulse generation is postulated to be suspended by a lack of kisspeptin secretion in mammals during the pre-pubertal period. Indeed, previous studies demonstrated a pubertal increase in *Kiss1* expression in rodents (42, 82) and a pubertal increase in kisspeptin secretion at the median eminence in primates (41).

Interestingly, the underlying mechanism of suppression of kisspeptin biosynthesis and secretion during pre-pubertal period differs from species to species. In rodents and sheep, ovariectomy acutely induced gonadotropin secretion even in pre-pubertal period, whereas estrogen replacement inhibited gonadotropin secretion until a normal pre-pubertal period expired (43, 82, 103). These findings suggest that estrogen plays a key role in the pre-pubertal restraint of GnRH/gonadotropin secretion and that the inhibitory action of estrogen seems to decrease during pubertal transition in those species (Figure 3). Indeed, estrogen strongly suppresses ARC *Kiss1* expression in female rats during the pre-pubertal period, but estrogen moderately suppresses ARC *Kiss1* expression in female rats during the post-pubertal period (82). Further, ovariectomy increased the number of ARC kisspeptin-immunoreactive cells in sheep in the pre-pubertal period, but not in the post-pubertal period (99). The regulation of ARC *Kiss1* expression by estrogen during pubertal transition is consistent with the classical “gonadostat hypothesis” (104, 105) which states that a decrease in the sensitivity to the negative feedback action of estrogen would be associated with the pubertal increase in GnRH/gonadotropin secretion in rodents. We envisage that a small amount of estrogen derived from the immature ovary directly inhibits *Kiss1* expression via estrogen receptor α (ERα) in kisspeptin neurons, because kisspeptin neuron-specific ERα knockout mice showed premature increases in ARC *Kiss1* expression and gonadotropin secretion, which resulted in the precocious pubertal onset (106, 107). Indeed, estrogen implantation into the ARC suppressed LH pulses in ovariectomized rats at the pre-pubertal period (43). Interestingly, estrogen implantation into the POA also suppressed LH pulses in ovariectomized rats at the pre-pubertal period, suggesting that ARC kisspeptin neurons are not a solitary inhibitory action site of estrogen to suppress *Kiss1* expression and hence GnRH/gonadotropin secretion in rats (43). Further studies are needed to clarify how sensitivity to estrogen decreases during pubertal transition in rats and sheep.

In primates, plasma gonadotropin profiles in gonadectomized animals were comparable to gonad-intact controls during juvenile and childhood: gonadotropin secretion was suppressed until just before puberty in both gonad-intact and gonadectomized animals (108, 109). Interestingly, estrogen-dependent suppression of gonadotropin secretion is manifested just before puberty onset in monkeys (110, 111). Thus, the central mechanism inhibiting GnRH/gonadotropin secretion in primates appears to differ from that in rodents and sheep (109, 112). A current interpretation for the brain mechanism controlling pubertal onset in primates was reported elsewhere (113). Briefly, two hypotheses have been suggested for the central suppression of GnRH/gonadotropin secretion during childhood to juvenile in primates (Figure 4). One is that tonic inhibition by γ-aminobutyric acid (GABA) neurotransmission would be responsible for the central suppression of GnRH/gonadotropin secretion in pre-pubertal monkeys (112); the other is that neuropeptide Y neurons could be responsible for the pre-pubertal suppression of GnRH/gonadotropin secretion (114). KNDy neurons may mediate the inhibitory role of GABA and/or NPY neurons in pre-pubertal restraint of GnRH neurons in primates. Further studies are needed to clarify how these inhibitory actions disappear at the pubertal onset in primates.

## Metabolic Control of Pubertal Onset

It has been well-accepted that nutritional or metabolic cues are important determinants of the initiation of tonic GnRH/gonadotropin secretion during pubertal transition, as evidenced by suppression of gonadotropin secretion when growth is retarded by a food restriction in several mammalian species including sheep (115) and rats (116, 117). Interestingly, such animals exhibited pubertal onset when they reached “critical” body weights, at which normally grown animals with *ad libitum* feeding exhibited pubertal onset (115, 116). The “critical” body weight hypothesis for determining pubertal onset was initially proposed by Frisch and Revelle (118) as an explanation of findings that girls showed puberty onset when they reached a body weight of around 47 kg at 17 years old in 1840s, and at 13 years old in 1960s along with nutritional improvements (119, 120).

Nutritional or metabolic cues reportedly control hypothalamic *Kiss1* expression in rodents. Castellano et al. (121) demonstrated that short-time fasting inhibited hypothalamic *Kiss1* expression along with the suppression of LH secretion in peripubertal rats. Inhibition of *Kiss1* expression and LH release by fasting has also been reported in adult rats and mice (122–126). Our recent tract-tracing study showed that hypothalamic kisspeptin neurons receive neuronal inputs from the hindbrain ependymocytes (127), which are suggested as a central energy sensor, in rats (128). The hindbrain ependymocytes, which express pancreatic glucokinase—a rate-limiting enzyme for glucose metabolism—and AMP-activated protein kinase, have been proposed to be able to sense, in particular, lowered glucose availability for controlling GnRH/LH secretion in rats (129–131). Thus, it is speculated that pre-pubertal suppression of hypothalamic *Kiss1*/kisspeptin expression might be, at least partly, mediated by this neuronal network.

Energy storage in the adipose tissue has been also considered to be involved in pubertal onset for a long time (132, 133). Leptin, the first hormone discovered to be secreted from the adipocytes, was then considered as a signal that relays the attainment of energy storage from the adipose tissue to the brain. Indeed, the leptin receptor is expressed in several hypothalamic and extra-hypothalamic nuclei including the ARC (134): KNDy neurons in the ARC express leptin receptors in mice (135). Importantly, leptin-null mice showed decreased ARC *Kiss1* expression (135) and pubertal failure (136). Moreover, leptin administration restored ARC *Kiss1* expression (135) and fertility in the leptin-null mice (136). Further, leptin administration induced pubertal onset in normal and growth-retarded rodents (137–140). These studies suggest that leptin is a prerequisite of normal pubertal onset. Interestingly, an increase in leptin secretion is not necessarily preceded with the pubertal onset in several mammals including primates and rodents (141–145). In humans, serum leptin concentration increased during pubertal development in girls, but remained constant in boys (141, 143). Circulating leptin remained constant during puberty in male monkeys (142, 144) and female rats (145). Donato et al. (146) showed that genetic deletion of the leptin receptor selectively from hypothalamic kisspeptin neurons had no critical effect on puberty and subsequent reproductive performance in mice, suggesting that leptin indirectly acts on kisspeptin neurons to control *Kiss1* expression. Further, recent studies suggested two separate leptin signaling pathways from ARC proopiomelanocortin (POMC) neurons and pituitary adenylate cyclase activating polypeptide (PACAP) neurons to ARC kisspeptin neurons in mice (147, 148). Overall, leptin is now considered as one of the permissive metabolic factors that allow pubertal development to proceed (149).

Ghrelin, a hormone mainly secreted from the stomach (150, 151), has been suggested to cooperate with leptin for the metabolic control of puberty onset in mammals (152–154): ghrelin has an inhibitory effect, while leptin has a stimulatory effect on pubertal onset. Indeed, repeated administration of ghrelin during pubertal transition partly delayed pubertal onset along with a decrease in serum LH and testosterone levels in male rats (155, 156). This notion is consistent with previous studies suggesting that ghrelin has an orexigenic effect as a peripheral signal of energy insufficiency, because ghrelin was shown to increase food intake in humans and rodents and plasma ghrelin levels increased by fasting and decreased by food intake in humans (157, 158). Further studies are needed to clarify the mechanisms underlying metabolic control of pubertal onset in mammals.

## Conclusions and Perspective

Studies during the twentieth century have increased our understanding of the mechanism controlling pubertal onset in mammals along with the discoveries of gonadotropins from the pituitary gland and GnRH from the hypothalamus. Most significantly, a breakthrough came in twenty-first century when kisspeptin and its receptor emerged. We now envisage that ARC kisspeptin neurons (also known as KNDy neurons), as a part of the GnRH pulse generator, are instrumental for pubertal onset in mammals via triggering GnRH/gonadotropin secretion. The timing of pubertal onset would be controlled by upstream regulators for kisspeptin biosynthesis and secretion. These are tightly controlled by steroid-dependent (rodents and sheep) or steroid-independent (primates) mechanisms as mentioned above. In addition, pubertal changes in kisspeptin biosynthesis and secretion may also be controlled by nutritional or metabolic cues. Further studies are needed to fully elucidate underlying mechanism of pubertal onset in mammals.

## Author Contributions

YU collected the information and wrote the manuscript. SN collected the information and designed the pictures. NI and HT collected the information and critically revised the manuscript.

### Conflict of Interest Statement

The authors declare that the research was conducted in the absence of any commercial or financial relationships that could be construed as a potential conflict of interest.
